# Experiential Knowledge Affects the Visual Search Behaviors of Sprint Coaches and Sport Biomechanists

**DOI:** 10.3389/fspor.2020.00095

**Published:** 2020-07-30

**Authors:** Amy Waters, Derek Panchuk, Elissa Phillips, Andrew Dawson

**Affiliations:** ^1^Institute of Health and Sport, Victoria University, Melbourne, VIC, Australia; ^2^Movement Science, Australian Institute of Sport, Canberra, ACT, Australia

**Keywords:** running, track and field, biomechanics, technique, verbal reports, visual pivot

## Abstract

It is common for applied sport biomechanists and high-performance coaches to work closely together. A feature of this relationship is that both bring unique experiences and knowledge to the common goal of improving an athlete's performance. For sprint running, coaches and biomechanists place importance on different aspects of technique. The purpose of this paper was to determine if these differences in experiential knowledge impact coaches and biomechanists visual perception of sprinting technique. Sport biomechanists (*n* = 12) and, expert (*n* = 11) and developing (*n* = 11) coaches watched video of athletes sprinting at two different speeds while wearing eye tracking glasses and, retrospectively, reported on the technique features observed. Mixed methods ANOVAs were used to determine visual search strategies and efficiency and used to indicate the relationship between visual search and verbal commentary data. The speed of video playback was the main determinant of visual search behavior, significantly impacting the visual search rate and relative fixation duration at a number of areas of interest. The use of a visual pivot indicated all participants' visual search strategies were efficiency driven. Overall, the verbal commentary did not completely align with the eye tracking data and there were varying degrees of agreement with the identified technique related areas of interest for coaches and biomechanists. However, differences in visual search strategy and verbal commentary suggest that experiential knowledge impacts participants' observation and perception of sprinting technique.

## Introduction

In high-performance sport, it is common for sport scientists, such as biomechanists, to work closely with coaches to improve an athlete's performance (Collins et al., 2015; Waters et al., 2019a). Sport biomechanists, employed by state institutes and academies of sport in Australia, develop a working relationship with sprint coaches and can play a role in changing an athlete's sprinting technique. This is generally done through the filming of performances at training and competitions and conducting detailed analyses that reveal how athlete technique is produced (Lees, 1999). This information allows a coach to make decisions about modifications to the athletes' running technique or their training program.

A key aspect of the coach-biomechanist relationship is the sharing of information and knowledge of sprinting technique and applying it to the athlete. Previously published research has found that sprint coaches and biomechanists have different understandings of what are the most important aspects of sprinting technique (Waters et al., 2019b). Sprint coaches emphasize arm and upper body movement (Waters et al., 2019b) whereas biomechanists place an emphasis on the underlying mechanics that control the movement of the lower body (e.g., ground reaction forces and muscle activations) (Waters et al., 2019b). These differing priorities can be explained by coach and biomechanists acquiring their knowledge of sprinting through different experiences. Coaches primarily develop technique knowledge through coaching experience and often their own athletic experience, they learn from other coaches through formal and informal methods such as attending workshops or mentoring (Reade et al., 2009). Biomechanists primarily develop their technique knowledge through formal education to the postgraduate level, conducting their own research into the biomechanics of sports skills with the goal of improving performance or reducing injury risks (Elliott, 1999). They can also develop experiential knowledge by working in regional and national sport institutes or academies with many coaches and elite athletes in a range of sports, as well as their own athletic experiences (York et al., 2014). The aim of this study is to determine if these differences in knowledge between coaches and biomechanists affect the way they perceive the skill of sprinting by comparing visual search behavior of both groups.

Generally, expert performers demonstrate superior perceptual-cognitive skill through the use of extensive domain-specific knowledge to extrapolate key information from their environment and, subsequently, make better, more efficient decisions (Mann et al., 2007). Due to the highly developed and detailed knowledge of sprinting that Biomechanists have displayed previously, they are assumed to exhibit visual search behavior characteristics of experts (Waters et al., 2019b). Eye tracking can provide insight into the mechanisms underlying perceptual-cognitive expertise by giving researchers access to what information is being perceived from the environment as well as how the information is perceived through investigations into the visual search strategies used. Visual fixations, or when the eye is stable, are the most common feature measured as these are when visual information from the environment is perceived. The fixation location identifies an area of importance and the fixation duration reflects the importance given to that location (Williams et al., 1999; Land, 2006). This visual information is combined with relevant knowledge to inform the decision-making of the expert. It is thought that variations in knowledge between experts and non-experts and experts in different domains, such as the differences between coaches and biomechanists, mean different visual information is extracted or the same information interpreted a different way, leading to a different decision being made.

While the perceptual-cognitive expertise of sport scientists, including biomechanists, has not been studied before, coaches' visual search strategies have been investigated in a small number of sports previously [e.g., (Moreno Hernández et al., 2006; Giblin et al., 2013; Robertson et al., 2017)]. In individual sports, such as gymnastics, comparisons between expert coaches and judges have shown equivocal visual search behavior; experts have shown both larger (Bard et al., 1980), smaller (Moreno Hernández et al., 2002) and similar (Imwold and Hoffman, 1983; del Campo and Espada Gracia, 2017) number of fixations compared to the less experienced participants. Despite inconsistencies in visual search behavior, expert gymnastic coaches and judges usually displayed more accurate decision-making around the subsequent scoring and judgement of the skill (Flessas et al., 2014; Pizzera et al., 2018). This shows the important role existing knowledge plays in the decision-making process for coaches; even if a superior visual search strategy cannot be defined, coaches still display their expertise by using experiential knowledge to inform their visual perception. Previous trends also highlight this need for a secondary, ecologically valid task other than eye tracking data to explore the connection between expertise, knowledge and visual search behavior (Williams and Ericsson, 2005). For coaches where the typical method of communicating technique related information and feedback is verbal, the most appropriate secondary task is verbal reports (Ford et al., 2009).

This study compared sprint coaches and applied sport biomechanists eye movement and verbal commentary while they observed video of athletes sprinting at two playback speeds. As no previous research has examined sprint coaches and biomechanists visual search behavior, we hypothesized that:

1. Expert coaches and biomechanists would exhibit a more efficient visual search pattern with a smaller number of fixations than developing coaches (Mann et al., 2007; Murray and Hunfalvay, 2017) and differences in visual search behavior would be greater for the faster videos.
2. Participants would spend more time looking at areas considered to be important for sprinting performance (i.e., longer relative fixation durations would match locations established as important to sprinting technique (Waters et al., 2019b).
3. Participants' visual search behavior would reflect information provided in their retrospective verbal commentary.

## Materials and Methods

### Participants

Twenty-two sprint coaches and 12 sport biomechanists were recruited to take part in this study. Potential coaches were invited to participate during their attendance at National Track and Field events and biomechanists were invited to participate during their attendance at the same events or at a sports biomechanics conference. All participants completed a questionnaire to establish their level of experience in coaching or sport science. The inclusion criteria for the sprint coaches included a minimum of 2 years' experience as a coach (Table 1) and at least an intermediate level of accreditation (Athletics Australia Level 2 Intermediate Club Coach—Sprint, Hurdles & Relay stream or equivalent). Inclusion criteria for the Biomechanists was a postgraduate degree in a relevant area (e.g., Master of Exercise Science) and published research in sprinting biomechanics or experience working with coaches and athletes in track and field as a sport biomechanist (Table 1). Coaches were divided into two expertise groups based on their responses to the questionnaire, using multiple criteria, this was to allow for better comparisons with the biomechanist groups' visual search and verbal data (Nash et al., 2012). Coaches were classified as expert if they had at least 10 years of experience coaching combined with a high-level coach accreditation (e.g., Level 3 or above) and consistent success with athletes at the national level or above. If they didn't meet at least two of those criteria, they were classed as a developing coach. Prior to data collection, ethical approval was granted by the university human research ethics committee and every participant provided written informed consent.

**Table 1:** Participant demographic information.

**Profession**	***n***	**Age (years)**		**Experience (years)**	
		**Mean**	**Standard deviation**	**Mean**	**Standard deviation**
Coach—expert	11	61.50	8.29	27.27	9.96
Coach—developing	11	39.40	7.68	9.60	4.32
Biomechanist	11	37.83	6.53	10.4	5.5
Track and field athlete—male	3	20.67	3.09		
Track and field athlete—female	3	22.33	3.40		
Rugby union player	4	25.5	2.5		

### Preparing Test Videos

Ten athletes (*n* = 10), of varying sprint abilities and backgrounds, were filmed sprinting to present to the coach and biomechanist participants. Four athletes were national-level male rugby union players (Table 1) and the remaining six were state and national-level track and field athletes. The male track and field athletes had a mean 100 m personal best time of 11.04 s (Standard Deviation = 0.43 s), while the female track and field athletes had a mean 100 m personal best time of 12.53 s (*SD* = 0.5 s). Rugby union is a sport where sprinting speed is crucial and sprinting sessions are a part of the players training schedule. The athletes were purposefully recruited with the aim of displaying variability of sprinting technique, allowing the participants opportunity to provide a variety of verbal comments.

After a self-directed general warm-up, each athlete completed three maximal 40–60 m sprints in a 110 m indoor athletics track while being filmed. Athletes' speed was measured in the last 10 m of the sprint effort using light gates (Smartspeed, Fusion Sport, Australia). The distance of each sprint effort was self-selected by the athletes to ensure they were not accelerating through the 10 m light gate zone. Athletes were filmed from the sagittal plane using 5 fixed Prosilica GE cameras (Allied Vision Technologies, Burnaby, Canada) capturing at 100 Hz. Cameras were strategically placed so there was overlap in the field of view, allowing the videos to be synchronized and stitched together. Video from the fastest sprint effort from each athlete was edited to show the athlete running at a constant speed, removing accelerations and decelerations. Athletes wore plain dark clothing to minimize chances of identification or distraction by the participants.

As biomechanists and coaches commonly use video for technique feedback, two speeds were chosen, through pilot testing, to closely reflect typical use (Mooney et al., 2016). The first speed was the original speed, to represent the observation of sprinting live. A second “slow motion” version of each clip where the playback speed was reduced to 10% of the original was also created. These two speeds also increased the task complexity which can emphasize expertise differences (Gegenfurtner et al., 2011). Using the 20 videos, playlists were created that randomized the order of video playback mixing original speed and slow motion together.

### Data Collection

For the experiment, participants observed the sprinting videos while wearing eye tracking glasses and verbally reporting on each athlete's technique. Participants were fitted with the eye tracker (Eye Tracking Glasses; SensoMotoric Instruments, Germany) and adjustments made to their seating position and the viewing screen position to ensure comfort before the three-point calibration procedure that is standard to the eye tracker software was conducted. They were calibrated and viewed the videos on a 13-inch tablet (Hewlett-Packard Elite x2, United States of America, Palo Alto). The calibration consisted of using a black screen with three white crosses at different edges of the screen to represent the entire viewing area. After calibration, participants viewed four familiarization videos and practiced the verbal responses with the opportunity to clarify any procedures with the researchers.

For testing, participants were shown 20 videos, while their eye movements were recorded. After viewing each video, they were asked to provide a verbal response to the statement “Please describe your assessment of this athlete's technique.” There was no time limit for the verbal responses and participants were encouraged to recall as much as possible, however, prompting and probing questions from the researcher were limited. Verbal responses were recorded using the microphone built into the eye tracking glasses. After the response was provided, a 3 s countdown prompted the beginning of the next video. Participants were given a short non-timed break after 10 videos to adjust their position or rest, and if required, re-calibration took place. The duration of each data collection session varied from 20 to 45 min, depending on the length of verbal responses and break taken after 10 videos.

### Data Analysis

Eye tracking data was analyzed using BeGaze (SensoMotoric Instruments, Germany) where individual fixations were identified, and fixation locations coded. Locations were coded as areas of interest: Arm, Arm (1), Head, Lower Leg, Lower Leg (1), Pelvis, Torso, Upper Leg, Upper Leg (1), Visual Pivot UB, Visual Pivot LB and Other. Visual Pivot locations are for upper or lower body only and not tied to a specific area outside the body and “(1)” denotes the limb behind the torso or pelvis not the left or right side. Inter-rater agreement for assessing fixation location was high (Cohen's kappa = 0.889) (Stuart et al., 2017).

Participants' data was averaged for each video condition, with the non-altered speed denoted as “Fast” and the slow-motion condition denoted as “Slow.” The visual search variables collected were number of fixation locations, number of fixations per second and fixation duration. For each location, relative duration was calculated as the percentage of total time spent fixating on that location relative to the length of the clip. A 3 × 2 analysis of variance (ANOVA) was used to determine the effect of expertise (between-group) and video speed (within-group) on visual search behavior, statistical significance was set at *p* < 0.05. Partial eta squared (ηp^2^) effect sizes were used to support the ANOVA results.

The verbal commentary audio of sprinting technique was coded using a framework adapted from swimming technique research (Rutt Leas and Chi, 1993). The adapted framework identified 30 items that included the fixation locations from the eye tracking data as well as concepts commonly used to describe sprint running technique, such as contact time and stride length (Table 2). The average number of verbal comments and percentage of features mentioned by each participant were included in the visual data ANOVA.

**Table 2:** Verbal commentary of sprinting technique analysis framework.

**Biomechanical principle**	**Area**	**Location/timing**
Kinematics	Posture	Head
		Shoulders
		Trunk
		Hips/pelvis
	Arms	Front arm
		Back arm
	Thigh	Late swing thigh
		Stance thigh
		Early swing thigh
	Knee	Late swing knee
		Stance knee
		Early swing knee
	Shin	Late swing shin
		Stance shin
		Early swing shin
	Foot	Late swing foot
		Stance foot
		Early swing foot
	Upper body	
	Lower body	
Kinetics	Braking	Impulse
		Peak
	Propulsion	Impulse
		Peak
Stride length		
Stride frequency		
Flight time		
Contact time		
Frontside mechanics		
Backside mechanics		

## Results

There was no significant expertise-based differences found, which does not support the first hypothesis. Therefore, results are reported and discussed in relation to video playback speed.

### Visual Search Efficiency (Hypothesis 1)

Video speed significantly affected the number of fixations [*F*_(2, 24)_ = 518, *p* < 0.05, ηp^2^ = 0.98] and fixation rate [*F*_(2, 24)_ = 1.633, *p* < 0.05, ηp^2^ = 0.12]. The average number of fixations for the fast video clips was 4.15 (*SD* = 0.95) and 20.93 (*SD* = 4.31) for the slow video clips. The average number of fixations per second for the fast video clips was 1.27 (*SD* = 0.28) and 1.36 (*SD* = 0.28) for the slow video clips.

However, the average fixation duration did not significantly differ in the two video speed conditions. For the biomechanists the average fixation duration for the fast and slow video clips were 511.68 ms (*SD* = 76.73) and 564.53 ms (*SD* = 94.63), respectively. The expert coaches were 466.52 ms (*SD* = 177.20) and 539.36 ms (*SD* = 187.8). The developing coach group 689.20 ms (*SD* = 293.98) and 652.69 ms (*SD* = 127.18).

The main determinant of visual search behavior appeared to be the speed of the video clip. In the “Slow” condition participants had more fixations and a higher search rate (fixations per second).

### Visual Search Locations (Hypothesis 2)

For several fixation locations there were significant differences between the relative fixation durations for the two video speeds (Table 3). For the fast clips more time was spent fixating on larger, central body segments such as the torso, pelvis and upper leg locations. There was also significantly more time spent at locations close to, but outside of the body. These locations were coded as visual pivot upper or lower body as they were located where multiple fixation locations could be seen (Kato and Fukuda, 2002). A common upper body visual pivot position was slightly in front of the athlete's torso, this allowed the participant to potentially perceive arm movement, and postural positioning of the torso and pelvis. A common lower body visual pivot position was in the space below the athlete's body where both legs would move through at some point of the sprinting gait cycle. From here participants could perceive the movement of the upper leg, extension of the knee and foot position in the lead up to contact with the ground. For the slow video clips, there was significantly more time spent fixating on smaller and faster moving segments such as the rear lower leg [lower leg (1)] and front arm (arm).

**Table 3:** Relative duration of fixations at each location by video clip speed.

**Location**	**Speed**	**Relative duration (%)**	***SD***	**Significance (*p*)**	**Effect size (ηp^**2**^)**
Arm	Fast	0.24	0.49	0.01^*^	0.228
	Slow	0.74	0.77		
Arm (1)	Fast	2.11	2.79	0.26	0.061
	Slow	1.56	1.04		
Head	Fast	3.35	5.17	0.06	0.142
	Slow	1.65	1.51		
Lower leg	Fast	1.72	2.93	0.15	0.084
	Slow	2.47	0.95		
Lower leg (1)	Fast	0.47	1.02	<0.01^*^	0.293
	Slow	1.15	1.04		
Other	Fast	0.57	1.01	0.04^*^	0.169
	Slow	1.00	1.15		
Pelvis	Fast	8.77	7.98	<0.01^*^	0.417
	Slow	2.65	1.42		
Torso	Fast	13.16	7.14	<0.01^*^	0.712
	Slow	3.21	1.73		
Upper leg	Fast	5.01	3.80	<0.01 ^*^	0.319
	Slow	2.95	1.18		
Upper leg (1)	Fast	1.38	1.82	0.90	0.001
	Slow	1.33	0.85		
Visual pivot LB	Fast	6.48	5.36	0.01^*^	0.280
	Slow	3.36	1.20		
Visual pivot UB	Fast	6.87	6.90	<0.01^*^	0.342
	Slow	2.82	1.56		

“*(1)” denotes the limb behind the torso or pelvis not the left or right side. LB, Lower Body; UB, Upper body; SD, Standard Deviation;*

* *denotes p < 0.05*.

Expertise did not significantly affect the relative duration of fixations at any of the 12 fixation locations. There was also no interaction between the speed and expertise variables, apart from the relative duration of fixations on the upper leg location. Developing coaches spent significantly less time fixating [*F*_(2, 24)_ = 3.39, *p* < 0.05, ηp^2^ = 0.22] on this location compared to the expert coaches and biomechanists during the Fast video clips.

### Verbal Commentary (Hypothesis 3)

The number of verbal comments was significantly affected by video speed [*F*_(1, 31)_ = 36.33, *p* < 0.05, ηp^2^ = 0.54]. The average number of comments for the fast video clips was 1.2 comments (*SD* = 0.55) and 1.86 comments (*SD* = 0.71) for the slow video clips.

As suggested by the visual search data, for the slow videos all participants mentioned a significantly high percentage of features, on average 40.4% (*SD* = 9.6%) of the 30 potential features were mentioned. [*F*_(1, 31)_ = 11.13, *p* < 0.05, ηp^2^ = 0.264]. Participants made significantly more comments related to the lower leg, lower leg (1), pelvis, upper leg and visual pivot upper body locations (Table 4). For the fast videos' participants mentioned, on average, 34.4% (*SD* = 12.9%) of possible sprinting technique features, with significantly more comments related to the head.

**Table 4:** Verbal comment count for each location by video clip speed.

**Location**	**Speed**	**Mean verbal comment (count)**	***SD***	**Significance (*p*)**	**Effect size (ηp^**2**^)**
Arm	Fast	3.07	2.59	0.16	0.08
	Slow	3.70	2.95		
Arm (1)	Fast	3.11	2.65	0.72	0.01
	Slow	3.30	3.14		
Head	Fast	0.67	1.00	0.03^*^	0.19
	Slow	0.26	0.71		
Lower leg	Fast	4.04	3.20	<0.01^*^	0.44
	Slow	7.74	4.28		
Lower leg (1)	Fast	0.96	1.58	0.01^*^	0.24
	Slow	2.41	2.68		
Pelvis	Fast	1.33	1.33	<0.01^*^	0.36
	Slow	3.63	3.70		
Torso	Fast	2.30	1.88	0.55	0.02
	Slow	2.04	1.63		
Upper leg	Fast	2.00	2.08	0.01^*^	0.24
	Slow	3.48	2.17		
Upper leg (1)	Fast	0.22	0.97	0.10	0.11
	Slow	0.78	1.67		
Visual pivot LB	Fast	5.11	3.49	0.13	0.09
	Slow	4.15	2.51		
Visual pivot UB	Fast	1.52	1.45	0.02^*^	0.20
	Slow	2.11	1.76		

“*(1)” denotes the limb behind the torso or pelvis not the left or right side. LB, Lower Body; UB, Upper body; SD, Standard Deviation;*

* *denotes p < 0.05*.

## Discussion

The aim of this study was to determine if experiential knowledge of sprint running technique affected participants' visual search behavior. Hypothesis 1 was that the expert coaches and biomechanists greater experience and knowledge of movement mechanics would direct their eye movement to more relevant areas and result in a smaller total number of fixations (Mann et al., 2007). This was not supported, as for both video speeds, expertise did not have a significant effect on the number, duration of fixations or the fixation rate. However, the speed of video playback did significantly impact participants' visual search behavior.

Video playback speed was the main determinant of visual search behavior. The average duration for video clips from the fast condition was 3.2 s and for the slow video clips it was 15.44 s. The fast video clips gave participants a lot less time to perceive the athlete's sprinting technique and required a different strategy to maximize the information taken in. This was exemplified by the participants fixating on the larger, slower moving areas of interest significantly more during the fast videos. The fast video condition saw the prevalent use of visual areas outside of the body where multiple body parts either rotated through or could be seen in the near periphery (i.e., a visual pivot). The presence of the visual pivot positions were an example of participants altering their visual search strategy and possibly relying on peripheral vision due to the time restricted nature of the task and to increase efficiency (Williams and Elliot, 1999; Savelsbergh et al., 2002). The locations and prevalence of the visual pivot points support sprinting technique areas of interest identified by both coaches and biomechanists and are evidence of all participants moving toward more efficient visual search behavior when under time pressure.

The second hypothesis was partially supported. As suggested by previous research (Waters et al., 2019b) it was expected that expert and developing coaches would fixate on the upper body, specifically locations related to posture and arm movement. This was the case for posture related locations (head, torso, pelvis) that had the longest relative fixation durations for both fast and slow video. The emphasis on these areas could also have been assisted by the segment's lack of movement, making them easier to fixate on as an athlete moved across the screen (Hutton et al., 1983; Huff et al., 2010). Despite coaches believing that arm movement is important to sprinting technique they did not fixate on the area as much as expected. This could again be due to speed of movement in the video, where tracking the fast-moving arm was difficult.

For the biomechanists it was expected they would fixate more on the lower body, with a greater emphasis on the lower leg around the late swing phase, immediately prior to the foot contacting the ground. This was not the case for both fast and slow video conditions with there being no emphasis on these locations. However, there was some weighting toward the lower body, with the lower body visual pivot location having a longer relative fixation duration for biomechanists than coaches. Although this does not reveal if specific phases of the sprinting gait cycle were fixated on more than others, so cannot determine if the visual pivot position was specifically used during the late swing phase of the sprint cycle. Unexpectedly, biomechanists had longer relative fixation durations for the postural locations in the slow video clips. This suggests that biomechanists may have been using a different visual search strategy to the coaches because when the time constraint of the fast video clip was removed, they chose to fixate on different areas with the extra time. Coaches generally added the faster moving segments of the lower leg and arms whereas the biomechanists added the postural locations. Potentially biomechanists' gap in knowledge in this area of sprinting technique means that they require more time to process posture related technique information and therefore cannot be as efficient as the coaches.

For the subsequent verbal commentary, the coaches made more comments about arm movement than posture in both video conditions, despite spending very little time, if any, visually fixating on the arms. The biomechanists verbal commentary matched the expected lower leg emphasis and despite fixating on posture related locations in the slow videos, this did not result in an increase in comments relating to the same locations. This could be due to the nature of that key technique feature. Ideal sprinting posture is defined by a lack of movement, therefore despite it being considered important and being a feature of all participants' visual search strategies, posture can only manifest as a very small number of verbal statements. Compared to arm movement where there are many possible technique features resulting in multiple comments. The results do not support the third hypothesis, as the verbal commentary does not reflect the visual search behavior found. However, verbal commentary matched the expected areas of interest better than the visual search data. This reflects the importance of existing knowledge in interpreting visual data (Kruijne and Meeter, 2016), potentially it was difficult to perceive technique changes in the videos and the participants had to rely on existing knowledge structures to make decisions about each athletes' technique and make the related verbal commentary rather than utilize the visual information to inform the verbal comments (Sherman et al., 2001; Robertson et al., 2017). This supports previous research where participants' results in the secondary task, judging gymnastic performance more accurately, were reflective of their expertise level despite inconsistent differences in the visual search data of novice and expert rhythmic and artistic gymnastic judges (Flessas et al., 2014; Pizzera et al., 2018).

Mental models of ideal technique are used as references by skilled observers such as coaches and biomechanists when observing technique in the daily training environment (Rutt Leas and Chi, 1993). The visual perception of technique is a crucial element of the technique analysis process (Sherman et al., 2001); therefore, the aim of this research was to determine if the knowledge differences found in previous research effect visual perception patterns when observing sprinting technique. It was anticipated that coaches would fixate on the upper body more than biomechanists, while biomechanists were expected to focus more on the lower body. Both the visual search and verbal commentary data suggest that expertise does not play a role in the observation of sprint running technique. Both the visual search and verbal commentary data suggest that expertise does not play a role in the observation of sprint running technique. Expert coaches did not significantly differ from developing coaches or sprint biomechanists, three groups that bring varied types and amount of experiential knowledge to the task. This could suggest that sprint coaches' expertise lays in other areas of coaching. Participants were found to utilize visual pivot positions to increase the efficiency of their visual search. Visual pivot's have not been found in a coaching population before, though there is some evidence in athlete populations that the use of peripheral vision is crucial when perceiving fast movements (Kato and Fukuda, 2002).

One limitation of this research is that the verbal commentary is unverified, as the results suggest that the verbal data reflected existing knowledge rather than the visual search data. Further investigation into the specific verbal comments made by participants and the biomechanics of the athletes may shed more light on this video observation skill that is a prevalent part of modern coaching. The error detection ability or visual sensitivity to changes in body positions of coaches and biomechanists are two potential directions that have already given insights into expert tennis coaches perceptual abilities and are potentially worth exploring in sprint coaches and biomechanists (Giblin et al., 2016). Another limitation of this research is the use of videos obtained in a controlled environment and displayed at fixed speeds, potentially collecting the visual and verbal data “live” in actual coaching sessions or during competition would reveal different insights into behavior. The slight changes in visual search strategy and verbal commentary suggest that in some way experiential knowledge impacts participants' observation and perception of sprinting technique. However, due to the inconsistencies between the visual and verbal data there is further investigation required to determine how this may affect the next phase of the coach-biomechanist relationship, the interaction and sharing of technique information to impact an athlete's performance.

## Data Availability Statement

The datasets generated for this study are available on request to the corresponding author.

## Ethics Statement

The studies involving human participants were reviewed and approved by VU Human Research Ethics Committee (VUHREC) Victoria University, Melbourne, Australia. The patients/participants provided their written informed consent to participate in this study.

## Author Contributions

AW collected data, conducted analysis with DP, and wrote first draft of manuscript. DP, EP, and AD contributed to manuscript revision and approved the submitted version. All authors contributed to the idea and design of study.

## Conflict of Interest

The authors declare that the research was conducted in the absence of any commercial or financial relationships that could be construed as a potential conflict of interest.
